# Novel Insights into Antiviral Gene Regulation of Red Swamp Crayfish, *Procambarus clarkii*, Infected with White Spot Syndrome Virus

**DOI:** 10.3390/genes8110320

**Published:** 2017-11-10

**Authors:** Shaokui Yi, Yanhe Li, Linlin Shi, Long Zhang

**Affiliations:** Key Lab of Freshwater Animal Breeding, Ministry of Agriculture, Key Lab of Agricultural Animal Genetics, Breeding and Reproduction of Ministry of Education, College of Fisheries, Huazhong Agricultural University, Wuhan 430070, China; yishaokui@foxmail.com (S.Y.); 376603966@webmail.hzau.edu.cn (L.S.); 15681195117@163.com (L.Z.)

**Keywords:** *Procambarus clarkii*, RNA-Seq, white spot syndrome virus, immune response

## Abstract

White spot syndrome virus (WSSV), one of the major pathogens of *Procambarus clarkii*, has caused severe disruption to the aquaculture industry of *P. clarkii* in China. To reveal the gene regulatory mechanisms underlying WSSV infection, a comparative transcriptome analysis was performed among WSSV-infected susceptible individuals (GS), viral resistant individuals (GR), and a non-infected control group (GC). A total of 61,349 unigenes were assembled from nine libraries. Subsequently, 515 and 1033 unigenes exhibited significant differential expression in sensitive and resistant crayfish individuals compared to the control group (GC). Many differentially expressed genes (e.g., *C-type lectin 4*, *Peroxinectin*, *Prophenoloxidase*, and *Serine/threonine-protein kinase*) observed in GR and GS play critical roles in pathogen recognition and viral defense reactions after WSSV infection. Importantly, the glycosaminoglycan biosynthesis-chondroitin sulfate/dermatan sulfate pathway was identified to play critical roles in defense to WSSV infection for resistant crayfish individuals by upregulating the chondroitin sulfate related genes for the synthesis of WSSV-sensitive, functional chondroitin sulfate chains containing E units. Numerous genes and the key pathways identified between resistant and susceptible *P. clarkii* individuals provide valuable insights regarding antiviral response mechanisms of decapoda species and may help to improve the selective breeding of *P. clarkii* WSSV-resistance.

## 1. Introduction

The red swamp crayfish, *Procambarus clarkii* (Girard 1985), native to north-eastern Mexico and south-central United States, is one of the world’s most invasive species [1]. In 1920s, *P. clarkii* was firstly introduced into Nanjing City, China from Japan [2] and is now widely distributed in almost all forms of the freshwater bodies [3]. Interestingly, it is widely favored and consumed in China, and is one of the most economically important farmed aquatic species rather than a devastating invasive species in China. In 2016, the total annual production of *P. clarkii* reached nearly 840,000 metric tons based on the Ministry of Agriculture data in China (www.yyj.moa.gov.cn). However, recently, with the rapid development of the aquaculture industry, various pathogens (e.g., bacteria, viruses, and rickettsia-like organisms) become the main constraint and have relatively increased the economic risks of artificial cultivation [4,5,6,7].

White spot syndrome virus (WSSV)—a rod-shaped virus infecting the cuticular epithelium [8], connective tissues, and hematopoietic tissues of crayfish [9]—has a broad host range within Decapoda crustaceans and is a serious pathogen in crayfish aquaculture [10]. Both farmed and wild *P. clarkii* in China have been natural hosts for WSSV which has caused severe disruption to the aquaculture industry of *P. clarkii* [11]. Invertebrates lack an acquired immune system but develop an innate immune system to defend against pathogenic microorganisms [12], and *P. clarkii* has also been used as a model organism to investigate the antiviral immunity of the invertebrate innate immune system [13,14]. Previous studies have reported many chemical and physical methods (i.e., oral administration of viral proteins, in vivo injection of inactivated WSSV, hyperthermia) that could be used to protect *P. clarkii* against WSSV [15,16,17], but these methods are still very difficult to be widely implemented in *P. clarkii* aquaculture. When hosts are challenged by pathogens, genes can be synergistically mobilized to play their respective roles in defense, particularly in the humoral immune response [18]. It is crucial to study immune-related gene functions in order to understand the coordination mechanisms of the innate immune system in *P. clarkii*.

Recently, RNA-seq technology has been widely used to explore the gene dynamic of crustacean species. Previously, Du et al. proposed that the Janus kinase/signal transducers and activators of transcription (JAK-STAT) signaling pathway might play an important role in intestinal antiviral immunity of *P. clarkii* based on RNA-seq data [19]. The lymph organ transcriptomes of normal and WSSV-challenged *P. clarkii* revealed that three potential antiviral signaling pathways (JAK/STAT, insulin, and Wingless-type (Wnt) signaling pathways) were involved in crayfish antiviral innate immunity [20]. Although several transcriptomes have been reported in the previous studies [6,19,20,21], the innate immune response of WSSV infected *P. clarkii* and the potential gene regulation involved in protection of defense to WSSV infection are still indistinct. Notably, a number of WSSV infected *P. clarkii* individuals exhibited viral resistance and survived in for long time. A comparative RNA-seq analysis would help uncover the genetic mechanism of *P. clarkii* WSSV-resistance, which would inform future aquaculture efforts. This study was designed to address the question: what is the superior regulatory mechanism of the WSSV resistant individuals? In this study, we utilized the RNA-seq approach to investigate the gene dynamics of three experimental groups, including WSSV-infected susceptible individuals, viral resistant individuals, and a non-infected control group. The gene expression patterns detected based on the RNA-seq data would be used to clarify the immune response of crayfish after infection and the genetic superiority of WSSV resistant individuals.

## 2. Materials and Methods

### 2.1. Experiment Design and Sample Collection

This study has been approved by the Institutional Animal Care and Use Committee (IACUC) of Huazhong Agricultural University (Wuhan, China) and conducted in accordance with ethical standards and according to the national and international guidelines.

Healthy *P. clarkii* weighing approximately 10–20 g were selected from the fish base in Huazhong Agricultural University (China). The sampled individuals were reared in water tanks with adequate aeration at 24–26 °C. These crayfish were fed with a commercial pelleted feed once a day until one week before the trial. Firstly, walking legs from randomly selected individuals were subjected to PCR assays to ensure that the individuals were WSSV-free before experimental challenge. The PCR primers (Forward primer 5′-TCACAGGCGTATTGTCTCTCCT-3′; Reverse primer 5′CACGAGTCTACCGTCACAACATC-3′) were designed based on the conserved region from WSSV genome (GenBank accession: AF369029). The PCR amplifications were performed in a 25 μL reaction volume containing 2.5 μL of 10× buffer (with Mg^2+^), 0.5 μL of dNTP(TaKaRa, Dalian, China), 0.5 μL of each primer (10 μM), 20.5 μL of water, 0.3 μL of Taq DNA polymerase (TaKaRa), and 0.3 μL of DNA template (100 ng/μL). The PCR program consisted of an initial denaturation at 94 °C for 10 min, followed by 35 cycles of 94 °C for 60 s, 55 °C for 45 s, 72 °C for 60 s, and a final extension at 72 °C for 10 min. The PCR products were determined by 1.5% agarose gel electrophoresis using DNA markers. Then WSSV was injected into the abdominal segment of 30 individuals with at the dose of 0.1 mL per individual with the concentration of 1.5 × 105 copies/μL. Additionally, the (1) negative control group (GC) was injected with 0.1 mL of 0.69% saline solution. After 24 h, the first three crayfish with loss of equilibrium were removed and immediately sampled as a susceptible group. With the extension of surviving time, more and more individuals with loss of equilibrium were continuously removed, and the last three individuals left were regarded as the resistant group. Thus, these WSSV infected individuals were divided into two groups: (2) non-sensitive or resistant (GR) and (3) susceptible group (GS). Three individuals from each group (i.e., susceptible group, resistant group, control group) were chosen to collect four tissues, including tail muscle, gill, hepatopancreas, and stomach. The tissue samples were frozen immediately in liquid nitrogen, and stored at –80 °C.

### 2.2. RNA Isolation and Sequencing

A total of 36 RNA samples from the four tissues of nine individuals were isolated using TRIzol reagent following the manufacturer’s instructions, and then genomic DNA was removed using gDNA eraser (TaKaRa). RNA quality and concentration were determined using a NanoDrop 2000 (Thermo Fisher Scientific, Wilmington, DE, USA) and an Agilent Bioanalyser 2100 (Agilent Technologies, Palo Alto, CA, USA), respectively. After quantification with Qubit 2.0 florometer (Life Technologies, Carlsbad, CA, USA), equal masses of total RNA from the four tissues for each individual were pooled and reversed into cDNAs with a random hexamer primer for sequencing as PE125 on Illumina HiSeq™ 2500 sequencer (Illumina, San Diego, CA, USA) in Oebiotech Co., Ltd. (Shanghai, China). For RNA-seq, a total of nine samples (three biological replicates per group) were sequenced.

### 2.3. De Novo Assembly and Annotation

The quality of raw reads was checked and visualized with FastQC program (version 0.11.5) [22], and the reads with adaptors and the low-quality reads were trimmed with NGS QC Toolkit [23]. Transcriptome de novo assembly was carried out with Trinity [24] using Kmer = 25. To acquire non-redundant unigenes as long as possible, TGI Clustering Tool (version 2.1) [25] was applied for further sequence splicing and redundancy removal. Subsequently, BLASTx (version v2.2.26) alignment (E-value cut-off = 10^−5^) with five public databases, including NR (NCBI non-redundant protein sequences), NT (NCBI nucleotide sequences), Swiss-Prot, KEGG (Kyoto Encyclopedia of Genes and Genomes), and KOG (euKaryotic Orthology Groups), was performed, and the best aligning results were used to decide sequence direction of unigenes.

### 2.4. Differentially Expressed Analysis

Bowtie2 program [26] was used to count the number of reads that mapped to the genes. The read counts of each sample were imported into the R package DESeq2 [27] for the differential expression analysis. Gene expression levels were estimated using FPKM values (expected number of fragments per kilobase of transcript sequence per millions base pairs sequenced), and the FPKM value was calculated based on basemean values. The *p* value was adjusted using *q* value (false discovery rate), and *q* value < 0.05 and |log_2_ (foldchange)| > 1 were set as the threshold for significantly differential expression. Gene ontology (GO) enrichment analysis of the differentially expressed genes (DEGs) was implemented by the GOseq package based on Wallenius non-central hypergeometric distribution [28]. The significantly enriched pathways were defined with FDR (false discovery rate) ≤ 0.05 using KOBAS 2.0 [29].

### 2.5. Quantitative Real-Time PCR

One microgram of RNA from each sample was reverse-transcribed to cDNA using a real-time PCR (RT-PCR) kit (TaKaRa). Subsequently, quantitative RT-PCR (RT-qPCR) was conducted with SYBR Green I Master Mix (TaKaRa). In total, 11 DEGs were selected to determine the expression levels. The primers are listed in Table S1. The β-actin gene was used as the internal standard gene, which has been widely used in previous studies [30,31]. RT-qPCR was performed in a total volume of 20 μL on the Light Cycler 480 system (Roche Applied Science, Indianapolis, IN, USA) according to the manufacturer’s instructions, and reaction conditions were 95 °C for 5 min, followed by 40 cycles of 94 °C for 15 s, 58 °C for 30 s, and 72 °C for 30 s. The relative expression was calculated based on the comparative C_T_ (ΔΔC_T_) method. Linear regression analysis and graph plotting was performed using R (www.r-project.org/).

## 3. Results

### 3.1. Sequencing, Assembly, and Annotation

After removing and trimming the low-quality reads, adaptors, poly-A tails, and reads containing >5% unknown nucleotides, a total of 696,821,756 clean reads were achieved from the nine libraries (Table 1). The GC contents ranged from 43.5–46.0%. Due to the absence of genome information of *P. clarkii*, the de novo assembly of the transcripts was performed. After the de novo assembly and removing redundancy, a total of 61,349 unigenes were obtained. The length distribution of all unigenes was between 301 and 33,907 base pairs (bp) with an average length of 1480 bp (Figure S1).

To investigate the gene function, all unigenes generated above were subjected to BLASTx searches against five public databases. As a result, 18,577 (30.28%), 16,591 (27.04%), 14,754 (24.05%), and 7592 (12.38%) unigenes had homologous sequences in NR, Swiss-Prot, KEGG, and KOG, respectively. Meanwhile, 15,592 (25.42%) unigenes were mapped to the GO items. Notably, a total of 19,999 (32.60%) unigenes were annotated in these databases, and 41,350 (67.40%) unigenes showed no homology to all sequences deposited in public databases. Additionally, for main species distribution whose distribution matched against NR database, the unigenes were annotated to the invertebrates; of these, 22.51% of the matched unigenes showed similarities with *Hyalella azteca*, followed by *Tetrahymena thermophila* (4.72%), and *Zootermopsis nevadensis* (2.94%).

### 3.2. Gene Regulation Patterns of *P. clarkii* after White Spot Syndrome Virus Infection

**Figure 1:**
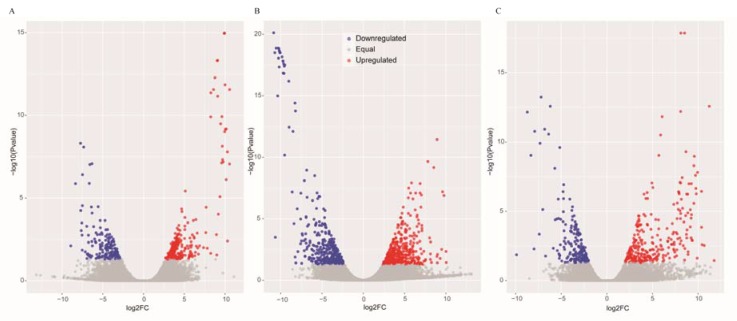
Scatter plots of the differentially expressed genes in GS vs. GC (**A**), GR vs. GC (**B**) and GR vs. GS (**C**). The blue scatters indicate the downregulated genes, and the red scatters indicate the upregulated genes.

**Figure 2:**
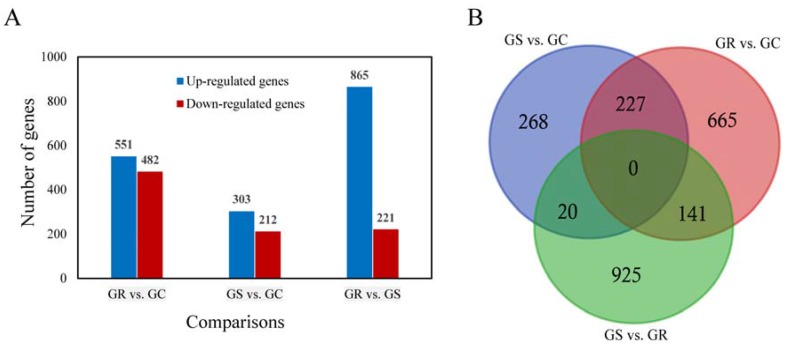
The number of differentially expressed genes (DEGs) from pairwise comparison in three *Procambarus clarkii* groups. The histogram graph showed the number of DEGs in each pairwise comparisons (**A**); Three-way Venn diagram of all evidencing numbers of the DEGs identified in the three comparisons (**B**).

The DEGs were identified by setting a criterion of >2-fold change in expression level (FDR < 0.05) between the groups with WSSV infection and control group. The differential expression of genes between the three groups was shown by plotting scatter plots (Figure 1). The number of DEGs is shown in Figure 2A and all DEGs can be found in Table S2. The numbers of DEGs identified in the GR vs. GC and GR vs. GS comparisons were approximately 2-fold greater than for that identified in the GS vs. GC comparison (Figure 2B). Among the genes that were found to be upregulated in GR compared to GS, several (e.g., *C-type lectin 4*, *peroxinectin*, *prophenoloxidase*, and *serine/threonine protein kinase*) are involved in various processes of immune response (Table 2).

### 3.3. Functional Categorization of Differentially Expressed Genes

GO terms were further assigned to the obtained unigenes based on their sequence similarities to known proteins. GO enrichment analysis showed that the genes involved in “Tie signaling pathway”, “cytokine activity”, “spinal cord patterning”, and “cell adhesion” were significantly downregulated in GS compared to GC (Figure S2A). The upregulated genes in GS vs. GC were significantly enriched in “translation” and “structural constituent of ribosome”. Notably, GO terms including “Tie signaling pathway”, “gluconolactonase activity”, “spinal cord patterning”, and “positive regulation of small GTPase mediated signal transduction” were enriched for the upregulated genes in GR vs. GC (Figure S2B), possibly indicating that genes related to these GO items are necessary for the antiviral response. Similarly, the downregulated genes in GR compared to GC were enriched in “translation”, “cytosolic large ribosomal subunit”, and “structural constituent of ribosome”.

To characterize the functional consequences of gene expression changes associated with the antiviral response and identify biological pathways activated in these three groups, the DEGs were classified based on the KEGG database. We compared the first 20 most enriched pathways among the three groups to identify those for genes related to antiviral response that were well represented for antiviral response. The DEGs downregulated in GS and upregulated in GR were significantly enriched in “Hematopoietic cell lineage”, “Glycosaminoglycan biosynthesis”, and “Other types of O−glycan biosynthesis”, etc. (Figure S3). Among the enriched pathways, “Glycosaminoglycan biosynthesis” (ko00532) was the most notable. Some genes downregulated in GS and upregulated in GR were significantly enriched in these pathways, which regulate multiple signaling pathways (e.g., Wnt, bone morphogenetic protein (BMP) signaling pathways, and fibroblast growth factor) and induce immune system upregulation by activating the contact system.

### 3.4. Glycosaminoglycan Biosynthesis Enhanced for Antiviral Response

**Figure 3:**
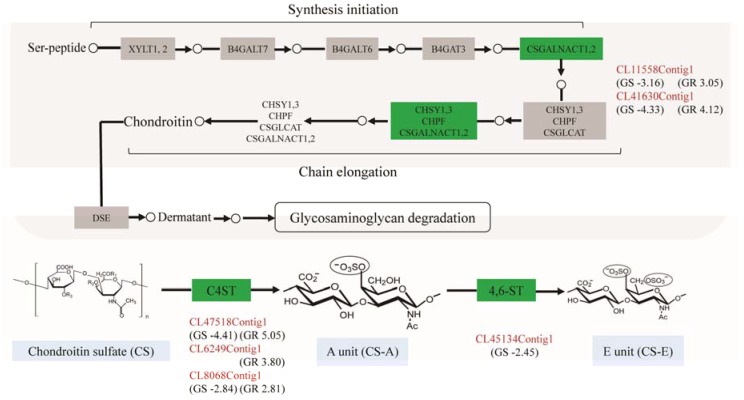
The pathway of glycosaminoglycan biosynthesis in *P. clarkii* and the related genes up- or downregulated in GS and GR. The unigene identity was colored in red; the green boxes represent the upregulated or downregulated gene(s); the numbers in the brackets indicate log2 (fold change).

The differentially expressed genes involved in glycosaminoglycan biosynthesis were listed in Table 3. These genes were downregulated in GS and concurrently upregulated in GR except *chst13* and *chst15*, which were not detected in the GS or GR (Figure 3). Of these genes, *csgalnact2* and *csgalnact1* play important roles in the elongation of chondroitin chains (upregulated in GR, 8.29 and 17.37-fold change; downregulated in GS, 8.95 and 20.13-fold change). Meanwhile, the other genes (*chst11*, *chst13*, *chst14* and *chst15*) involved in the synthesis of CS-A (GlcA-4SGalNAc) and CS-E (GlcA-4S, 6SGalNAc) were also differentially expressed in GS and GR. Notably, *chst13* was upregulated in GR and not differentially expressed in GS vs. GC (2.52-fold change; *p* > 0.05). Also, *chst15* was downregulated in GS, and no significant difference of expression level was observed in GR vs. GC.

### 3.5. Validation of the Differentially Expressed Genes

**Figure 4:**
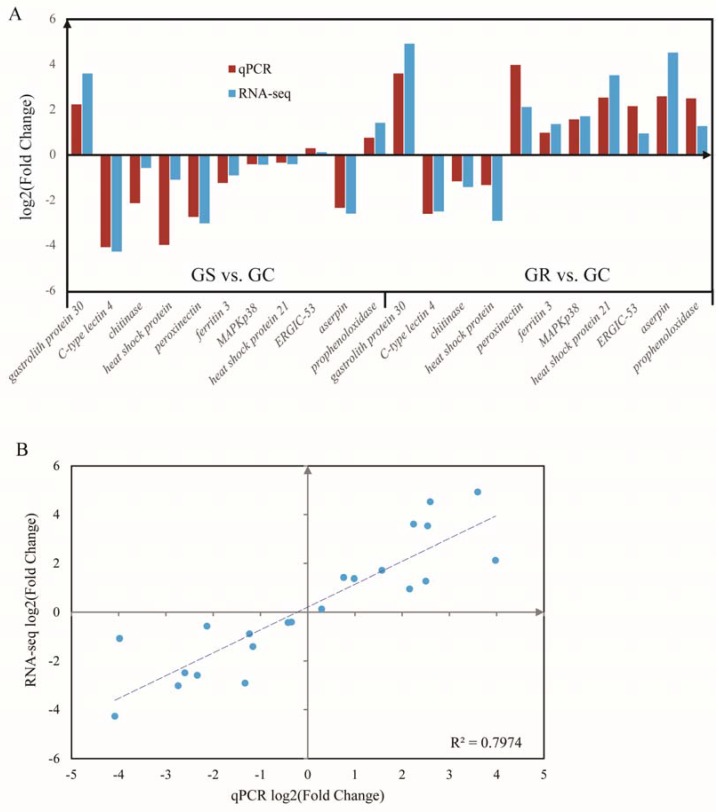
Quantitative real-time PCR (qRT-PCR) validation (**A**) and linear regression analysis (**B**) of RNA sequencing of selected DEGs.

RT-qPCR analysis was performed to validate the results of differential gene expression obtained from RNA-seq data. A total of 11 unigenes were selected for RT-qPCR analysis in this study. The results of qRT-PCR analysis coincided with the results generated from high-throughput sequencing (Figure 4A), and showed a similar expression trend with the RNA-seq data. Linear Regression of log2 (fold change) between RT-qPCR data and RNA-seq data were consistent with the correlation coefficient of 0.80 (*p* < 0.01) (Figure 4B).

## 4. Discussion

WSSV has been proven to infect and cause mortality and morbidity in shrimp or crayfish [9,11]. Due to the huge economic losses caused by WSSV on commercial crayfish farming in China, the need for WSSV-resistant domesticated stocks of *P. clarkii* is quite urgent presently. However, the transcriptional regulation underlying resistance to WSSV in crayfish is still largely unknown. Recently, several studies on the transcriptional responses after WSSV infection in crayfish have been documented [6,19,20]. These studies identified several important pathways involved in immune response of crayfish after WSSV infection, including the JAK-STAT, insulin, and Wnt signaling pathways. However, the individual genetic difference of resistance to WSSV was ignored, and also the gene regulation network for antiviral immune responses of crayfish is still ambiguous. As an attempt to reveal the genetic basis for the viral resistance in crayfish, a comparative transcriptome analysis between susceptible individuals and resistant *P. clarkii* individuals was performed in this study.

### 4.1. Immune Related Gene Enhanced in Resistant Crayfish after WSSV Infection

In this study, 515 and 1033 genes, respectively in susceptible and resistant individuals, were identified to be differentially expressed and associated with WSSV resistance. To these differentially expressed genes were assigned multiple potential functions in biological processes and cellular components. Here, we mainly focused on the immune-related genes that potentially mediated the viral resistance. WSSV resistance resulted in severe transcriptional upregulation of genes including calcium-dependent (C-type) lectin 4 (*ctl4*), peroxinectin, and serine/threonine-protein kinases (*stk*). Of these, *ctl4* plays important roles in innate immunity, cell–cell interactions, and especially in pathogen recognition in crayfish and has been reported to be involved in defense against bacterial and viral pathogens as pattern-recognition receptors (PRRs) [32]. As a cell adhesion protein, the mechanism of peroxinectin may act as the strong adhesion to the foreign targets forming a multilayered sheath of cells during encapsulation in crustacean cellular defense reaction. Meanwhile, *stk* is critical for the regulation of immune responses and is associated with signal transduction of pathogen recognition. These emerging gene regulation patterns reveal a genetic mechanism of pathogen recognition in WSSV infected crayfish.

Prophenoloxidase (*proPO*) was significantly upregulated (4.81-fold change, *p* = 0.01) in resistant individuals compared to susceptible individuals in this study. ProPO is an inactive form and is converted to the active form phenoloxidase (PO) after limited proteolysis by serine proteinases, which is the terminal component of the prophenoloxidase activating system [33,34]. The proPO-system is considered to be an important innate immune defense system in many invertebrates, notably arthropods [35]. The proPO-activating roles in the immune defense reactions were reported in crayfish species, including *P. clarkii* [36,37], *Pacifastacus leniusculus*, and *Astacus astacus* [38]. Conceivably, the antimicrobial compounds produced by the phenoloxidase activity play a critical role in WSSV resistance of resistant *P. clarkii* individuals. Ai et al. [39] reported that *proPO* was downregulated in *Litopenaeus vannamei* challenged with WSSV, suggesting it may be pivotal in the defense against WSSV. Similar results were also observed in Sookruksawong et al. [40]. Apparently, the roles of proPO system in viral resistance of crayfish need to be given more attention.

### 4.2. Glycosaminoglycan Biosynthesis Involved in Antiviral Response of Crayfish

We found that the genes differentially expressed in GS and GR were significantly enriched in glycosaminoglycan biosynthesis pathway, which regulate multiple signaling pathways, such as fibroblast growth factor (FGF)/FGFR, hepatocyte growth factor, vascular endothelial growth factor (VEGF)/VEGFR, platelet derived growth factor (PDGF)/PDGFR, Wnt, and BMP signaling pathways, where genetic studies have revealed an absolute requirement for glycosaminoglycan in these pathways [41]. Glycosaminoglycan polysaccharides have two major types, heparan sulfate and chondroitin sulfate [42]. Chondroitin sulfate (CS) is the principal pericellular and extracellular components that form regulatory milieu involving numerous biological and pathophysiological phenomena [43]. The disaccharide units of CS chains are classified into O, A, C, D, and E units on the basis of their sulfation patterns.

Viruses have been shown to utilize cell surface CS chains to attach to and infect host cells [43,44]. For instance, the adhesion of hemocytes infected by WSSV to endothelial cells requires CS chains bearing a low sulfated CS-A structure. CS chains rich in E units also serve as the cell surface receptors in the case of virus infection [45]. A previous study [46] has proven that the susceptibility to viral infection is reduced in murine sog9 cells that are deficient in chondroitin-4-sulfotransferase-1 (*C4ST-1*) expression and production of E units. Here, it could be inferred that the upregulation of CS related genes in resistant crayfish individuals is essential for the synthesis of WSSV-sensitive, functional CS chains containing E units, which are vitally important for crayfish to defend against WSSV infection.

Overall, this study provides a comparative transcriptome analysis between WSSV-resistant and WSSV-sensitive crayfish individuals. Many immune related genes and the glycosaminoglycan biosynthesis pathway were found to play critical roles in the antiviral response of *P. clarkii* to WSSV. The novel findings would contribute to an improved understanding of antiviral response of *P. clarkii* and help us improve the selective breeding of WSSV-resistant crayfish varieties.

## Figures and Tables

**Table 1:** The sequencing information from nine RNA-seq libraries.

Group	Sample	Raw Reads	Clean Reads	Clean Bases (Gb)	Valid Ratio (base)	GC Content (%)
GC	G1_E22	85,792,014	84,216,344	9.80	98.11%	46.00
	G1_E23	83,021,208	81,364,944	9.47	97.95%	46.00
	G1_E25	95,650,310	93,818,350	10.92	98.03%	46.00
GR	G2_E31	76,276,920	74,917,080	8.72	98.16%	44.00
	G2_E39	80,384,048	78,772,012	9.17	97.94%	45.00
	G2_E40	67,638,948	66,384,842	7.72	98.09%	44.50
GS	G3_E6	81,888,888	80,253,434	9.34	97.95%	44.00
	G3_E8	67,330,090	65,977,146	7.68	97.93%	43.50
	G3_E11	72,882,090	71,117,604	8.27	97.52%	45.00

GC, non-infected control group; GR, viral resistant individuals; GS, white spot syndrome virus (WSSV)-infected susceptible individuals.

**Table 2:** The candidate genes involved in the antiviral response for *P. clarkii*.

Unigene	Fold Change (GS/GR)	Log2FC	Annotation	FDR
CL41502Contig1	9.4600	3.2426	C-type lectin-2 (*Litopenaeus vannamei*)	0.0298
CL19887Contig1	4.9525	2.3081	C-type lectin 4 (*Litopenaeus vannamei*)	0.0071
CL8034Contig1	3.7954	1.9243	innexin inx2 (*Homarus gammarus*)	0.0015
CL46545Contig1	0.2819	−1.8267	serine proteinase inhibitor (*Procambarus clarkii*)	0.0028
CL29567Contig1	0.2568	−1.9611	serine protease (*Scylla paramamosain*)	0.0024
CL26397Contig1	0.2136	−2.2273	serine proteinase-like protein 1 (*Pacifastacus leniusculus*)	0.0006
comp98966_c2_seq4_1	0.1704	−2.5531	Serine/threonine protein kinase	0.0416
CL3961Contig1	0.3847	−1.3783	MAP kinase-interacting serine/threonine-protein kinase 1 (*Daphnia magna*)	0.0191
comp120608_c2_seq2_2	0.3304	−1.5976	mitogen-activated protein kinase p38 (*Scylla paramamosain*)	0.0080
CL31899Contig1	0.2222	−2.1703	peroxinectin (*Pacifastacus leniusculus*)	0.0042
CL15404Contig1	0.3836	−1.3821	integrin (*Pacifastacus leniusculus*)	0.0319
CL31391Contig1	0.2081	−2.2647	prophenoloxidase (*Pacifastacus leniusculus*)	0.0115
CL29721Contig1	0.0551	−4.1813	DnaJ domain protein (*Trichuris suis*)	0.0081
CL42408Contig1	0.1135	−3.1388	heat shock protein (*Cherax destructor*)	0.0473
CL47461Contig1	0.0701	−3.8347	heat shock protein 21 (*Macrobrachium rosenbergii*)	8.53 × 10^−6^
CL44350Contig1	0.1803	−2.4717	insulin-like growth factor binding protein 7-like protein (*Cherax quadricarinatus*)	0.0002
CL45485Contig1	0.1612	−2.6327	serpin8 (*Litopenaeus vannamei*)	0.0044
comp104708_c1_seq2_1	0.4205	−1.2497	ubiquitin (*Pygocentrus nattereri*)	0.0284
comp125239_c0_seq4_2	0.1409	−2.8269	aserpin	0.0012
comp94348_c1_seq1_3	0.2895	−1.7884	ferritin 3 (*Eriocheir sinensis*)	0.0267
CL3118Contig1	2.5278	1.3379	chitinase (*Pandalopsis japonica*)	0.0238

GR, viral resistant individuals; GS, white spot syndrome virus (WSSV)-infected susceptible individuals; FDR, false discovery rate.

**Table 3:** The differentially expressed genes involved in glycosaminoglycan biosynthesis.

Unigene ID	Gene Name	Gene Description	log2FC (GS vs.GC)	log2FC (GR vs.GC)	KO Entry
CL11558Contig1	*csgalnact2*	Chondroitin sulfate N-acetylgalactosaminyltransferase 1	−3.06	3.15	K00746
CL41630Contig1	*csgalnact1*	chondroitin sulfate N-acetylgalactosaminyltransferase 2	−4.33	4.12	K00746
CL47518Contig1	*chst11*	Carbohydrate sulfotransferase 11	−4.41	5.05	K01017
CL6249Contig1	*chst13*	Carbohydrate sulfotransferase 13	NA	3.80	K01017
CL8068Contig1	*chst14*	carbohydrate sulfotransferase 14	−2.84	2.81	K01017
CL45134Contig1	*chst15*	Carbohydrate sulfotransferase 15	−2.45	NA	K08106

KO, KEGG Orthology; NA, not available.

## Supplementary Materials

The following are available online at www.mdpi.com/2073-4425/8/11/320/s1, Table S1: The primer sequences of the selected genes for qRT-PCR, Table S2: The list of differentially expressed genes identified in the pairwise comparisons, Figure S1: The length distribution of the assembled unigenes, Figure S2: The GO enrichment of the DEGs in GS vs. GC and GR vs. GC, Figure S3: The enriched KEGG pathway in GS vs. GC and GR vs. GC.
